# Berzosertib enhances the sensitivity of pediatric diffuse midline glioma H3K27-altered cells to radiotherapy

**DOI:** 10.1038/s41419-026-08567-7

**Published:** 2026-03-20

**Authors:** Nikita Gorainow, Felix Sander, Daniel Picard, Marvin Christopher Frölich, Katharina Eul, Sarah Etemadi Afshar, Julia Asche, Michelle Monje, Eric Raabe, Jasmin Bartl, Arndt Borkhardt, Guido Reifenberger, Nicole Dünker, Maike Busch, David Pauck, Nan Qin, Johann Matschke, Marc Remke, Verena Jendrossek

**Affiliations:** 1 Institute of Cell Biology (Cancer Research), University of Duisburg-Essen, University Hospital Essen, Essen, Germany; 2 Department of Pediatric Oncology, Hematology and Clinical Immunology, Medical Faculty, University Hospital Düsseldorf, Düsseldorf, Germany; 3 Institute of Neuropathology, Heinrich Heine University Düsseldorf, Medical Faculty, University Hospital Düsseldorf, Düsseldorf, Germany; 4 Department of Pediatric Hematology and Oncology, University Medical Center of Saarland, Saarland University, Homburg, Germany; 5 Department of Neurology and Neurological Sciences, Stanford University, Stanford, CA USA; 6 Department of Oncology, Johns Hopkins University, Baltimore, MD USA; 7 Center for Translational Neuro- and Behavioral Sciences, Institute of Anatomy II, Department of Neuroanatomy, Medical Faculty, University of Duisburg-Essen, Essen, Germany; 8 Department of Hematology, Medical Faculty, Heinrich Heine University Düsseldorf, Düsseldorf, Germany; 9 Spatial & Functional Screening Core Facility, Medical Faculty, Heinrich Heine University, Düsseldorf, Germany; 10 German Cancer Consortium (DKTK) partner site Essen, a partnership between DKFZ and University Hospital Essen, Essen, Germany; 11 West German Comprehensive Cancer Center Essen (CCC-WTZ), Essen, Germany

**Keywords:** Paediatric cancer, Paediatric cancer

## Abstract

Diffuse midline glioma H3K27M-altered (DMG) remains a fatal pediatric brain cancer driven by a global loss of histone H3K27 trimethylation. Radiotherapy comprises the most important treatment modality and significantly improves overall survival. Novel therapeutic strategies for DMG patients without or with radiotherapy are urgently needed. Here, we aimed to gain insights into potential radiation response modulators. To identify modulators of radiation response, we performed a high-throughput drug screening (HTS) in seven representative DMG cell lines using conventional chemotherapeutic drugs and phase I-IV drugs (*n* = 687), followed by irradiation with 0 or 2 × 4 Gray (Gy). The ataxia–telangiectasia and Rad3-related (ATR) inhibitor berzosertib emerged as a potent radiosensitizer. Its effects were validated in three DMG cell lines using short-term proliferation assays, long-term limiting dilution assays (LDA), 3D spheroid cultures, and the chorioallantoic membrane (CAM) assay *in ovo*. Across all three tested DMG cell line models, berzosertib enhanced the antineoplastic effects of clinically relevant radiation doses, significantly reducing proliferation and clonogenic survival, delaying spheroid growth, and suppressing tumor formation *in ovo*. These findings provide strong preclinical evidence that ATR inhibition increases the sensitivity of DMG cells to radiotherapy. They highlight a novel therapeutic vulnerability and support further exploration of ATR inhibitors in rational combination strategies to improve radiotherapy efficacy for this deadly disease.

## Introduction

The WHO Grade IV diffuse midline glioma histone *H3K27-altered* (DMG), formerly known as diffuse intrinsic pontine glioma (DIPG), is a leading cause of cancer-related deaths amongst children and adolescents diagnosed with gliomas [1, 2]. So far, DMG remains a largely non-curable disease with a two-year survival rate of less than 10% [1–3] and an average survival time of less than one year after diagnosis [2, 4], respectively. Though DMG may also occur in the adult population, the disease is mostly diagnosed at ages between 7 and 9 years [1, 2]. While diagnosis can usually be inferred using typical MRI features, several studies recommend the upfront collection of biopsies for histological and molecular assessment [3, 4]. In fact, oncogenic drivers have been identified using stereotactic biopsies for molecular and histological assessment. Nowadays, diagnosis of DMG requires immunohistochemical prove of H3K27 hypomethylation (loss of H3 p.K28me3 (K27me3)) that is either associated with a point mutation in one of the H3 isoforms (*H3K27M)* (78% of the cases), overexpression of *enhancer of zeste inhibitory protein* (*EZHIP*), both negative regulators of polycomb repressor protein 2 (PRC2), pathogenic point mutation or amplification of the *epidermal growth factor receptor* (*EGFR*) [5, 6] or a methylation profile consistent with of one of the DMG subtypes. Other recurrent mutations that are frequently detected affect the tumor suppressor genes *TP53* [7] or *ataxia telangiectasia mutated* (*ATM*) [8, 9].

Despite breakthroughs in the molecular understanding of the oncogenic drivers of the disease the clinical management of DMG patients has not significantly changed in the past decade: Due to the intricate location of DMGs in the midline of the central nervous system, specifically brainstem/thalamus/spinal cord, and its diffuse and infiltrative growth pattern [10] surgical debulking or complete resection is not feasible. Furthermore, curative chemotherapeutic approaches have not been established thus far, though numerous chemotherapeutic approaches, targeted therapies, or immunotherapeutic approaches have been evaluated [11, 12, 13]. So far, only aggressive radiotherapy can provide a transient symptomatic improvement and a modest survival benefit [14]. The standard radiation regimen consists of administering a cumulative radiation dose of 54–60 Gray (Gy) in a six-week period [15]. This radiotherapeutic approach delays tumor progression for approximately three to six months [16, 17].

**Fig. 1:**
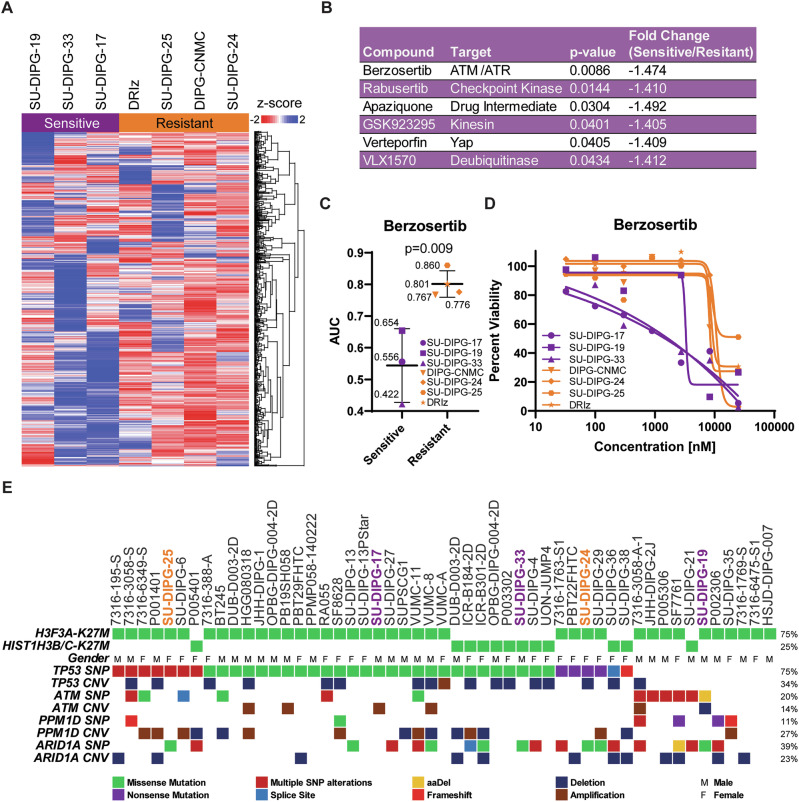
High-throughput screening identified enhanced antineoplastic effects of radiotherapy in combination with the ATR inhibitor berzosertib in DMG models in vitro. Heatmap showing the area under the curve (AUC) results from the high throughput screening of seven DMG cell lines after a total dose of 8 Gy (2 × 4 Gy) irradiation, divided into Sensitive and Resistant cell lines. Data are represented as z-scores of the normalized AUC values, and the dendrogram on the right represents relatedness of drugs to each other based on hierarchical clustering based on similarity in their AUC profiles across the seven cell lines. **A** Supervised analyses were performed and filtered for the following: *p* value < 0.05, fold change ±1.4 and not significant in the non-irradiated comparison (**B**). The AUCs of the seven cell lines for berzosertib, the top compound based on *p*-value, were displayed with a dot plot (**C**) and dose-response curves were plotted to show increased potency in the Sensitive group (**D**). Oncoprint of DMG cell lines showing key mutations in the characteristic histone genes and genes involved with DNA damage (TP53, ATM PPM1D and ARID1A) [8]. Cell lines used in the HTS are highlighted according to their respective groups (**E**).

Since radiotherapy is the only established therapeutic option to improve survival in this fatal disease to date, we aimed at the identification of radiosensitizing drugs to improve the treatment effects of the most effective current treatment modality. Various clinical trials have evaluated radiosensitizers for brain tumors including DMG [18–21]. Here, we applied an in vitro high-throughput drug screening (HTS) combined with a radiation setup in cell line models representative of DMG (Fig. 1E) and subsequently validated the most promising drug candidates in combination with radiotherapy in three DMG cell lines that capture both the molecular and phenotypic diversity of the disease. Specifically, HTS of DMG cell lines in combination with irradiation allowed us to identify the *ataxia telangiectasia and rad3-related protein* (*ATR*) inhibitor berzosertib as a promising radiosensitizer in DMG cell lines. The strong combinatorial effect of berzosertib with irradiation observed in the screening and the existing clinical experience with this drug generated in phase I-II studies encouraged us to validate this result in short- and long-term assays in vitro and in the chorioallantoic membrane (CAM) model *in ovo*. In these assays, berzosertib enhanced the antineoplastic effects of ionizing radiation on cell proliferation, clonogenic survival (limiting dilution assay), and spheroid growth, as well as *in ovo* (CAM model), respectively. Thus, HTS is suitable to characterize novel pharmacological vulnerabilities enhancing the effect of radiotherapy in DMG. Additionally, our data indicate that ATR inhibition may constitute a promising radiosensitizing strategy to achieve improved treatment responses to radiotherapy in DMG patients. Further validation of the obtained findings in orthotopic models in vivo and future clinical trials is required.

## Materials and methods

### Cell culture

The experiments of this paper have been performed using the human DMG cell lines SU-DIPG−17, −19, −24, −25 and −33 [22], kindly provided by the research team of Michelle Monje at the University Hospital Stanford and cell lines DRIz and CNMC from the working group of Eric Raabe at the University of Zürich. All cell lines have tested negative for mycoplasma. The cells were grown in serum-free medium containing 1:1 neurobasal-A and DMEM/F-12 medium (+ HEPES, - glutamine), 1x MEM non-essential-amino-acids, 0,5x glutamax, 1x sodium pyruvate, 1x sodium bicarbonate, 1x B27 minus vitamin A, 100 μg/ml penicillin/streptomycin (all from ThermoFisher, USA), 10 ng/ml PDGF-AA, 10 ng/ml PDGF-BB (Shenandoah Biotechnology, USA), 20 ng/ml bFGF (ThermoFisher, USA), 20 ng/ml EGF (ThermoFisher, USA) and 2 μg/ml heparin (StemCell Technologies, Germany).

### Drug screening for radiosensitizers

The drug library used to identify possible interactions of radio- and chemotherapy consisted of 687 anti-cancer drugs (MedChemExpress, USA). Every compound was used at concentrations ranging from 0 to 10 µM covering six to eight concentration levels. The drugs were printed into 1536-well microplates using the D300e digital dispenser (Tecan, Austria) and stored frozen after preparation. After sufficient numbers of the DMG cell lines were grown, the plates were thawed and filled with cell suspension using the MultiDrop Combi (ThermoFisher, USA) at 4 µl/well. Seeding density was optimized prior to seeding for each cell line. The Gulmay RS225 cabinet irradiator, equipped with a 0.2 µm copper filter, was used during the screening process for irradiating the microplates. For irradiation, we used 15 mA and 150 kV as device parameters. After a total incubation period of 72 h starting at the time of cell seeding, the drug/radiation response was measured by applying 4 µl of the luminescence-based ATP assay CellTiter-Glo (Promega, USA) per well and reading the microplate in a Spark microplate reader (Tecan, Austria). We calculated normalized area under the dose-response curve (AUC) data, as described in Qin et al. [23], and compared control screens (HTS in the context of mock irradiation) and combined treatment (HTS together with irradiation). Additionally, we performed a PCA using the top 10% most variable compounds from the screening results of drug treatment alone, selected based on their standard deviation, which is a commonly applied strategy to reduce noise in high-dimensional drug-response datasets.

### Irradiation to validate HTS findings

For radiobiological validation experiments, irradiation was performed as described elsewhere [24–26]. Briefly, cells were irradiated at room temperature with an X-ray machine (RS320, Xstrahl Ltd., UK) operating at 300 kV, 10 mA and a dose rate of 0.9 Gy/min using a rotating table. For validation of berzosertib effects, berzosertib was obtained from MedChemExpress, USA, and irradiation was performed 2 h after drug treatment, according to our established radiosensitization treatment protocol [25, 26].

### Proliferation assay

Cells were seeded in three different 96-well plates at 7.500 cells/well for each cell line. Cells were treated 16 h later with at least 10 different concentrations of berzosertib, ranging from 80 to 1800 nM and irradiated 2 h later, according to our established radiosensitization treatment protocol [25, 26]. Cell proliferation was measured using the CellTiterGlo assay 72 h after irradiation, which was performed according to the manufacturer’s instructions. The resulting luminescence signal was measured with the SYNERGY H1 ELISA reader (Agilent Biotek, USA).

IC50 and IC20 values were calculated by fitting nonlinear regression curves (log(inhibitor) vs. response, variable slope) to the dose-response curves generated from the proliferation assay data using GraphPad Prism 7.3 (GraphPad Software, USA). For each cell line, separate dose-response curves were generated under 0, 2, and 4 Gy irradiation. The IC50 was defined as the concentration producing 50% of the maximal inhibitory effect of berzosertib, and the IC20 as the concentration achieving 20% of the maximal inhibitory effect, based on the fitted dose-response curve. These values were used to determine concentration ranges for synergy analysis and to select the IC20 under 4 Gy irradiation as the reference concentration for subsequent long-term assays. Bliss-synergy scores were determined with SynergyFinder-Plus (https://synergyfinder.fimm.fi, [27]).

### Limiting dilution assay

Long-term survival of clonogenic DMG cells upon irradiation was assessed employing a limiting dilution assay as described elsewhere [28–30]. In brief, cells were seeded on a 96-well plate in tumor-serum-free-medium at increasing numbers from 2 to 512 cells per well. Each plate was treated with one concentration of berzosertib or temozolomide (33.3%*IC20, 66.7%*IC20, 100%*IC20) or DMSO control 16 h after cell seeding and irradiated 2 h after berzosertib treatment. Plates were incubated for 14–21 days after irradiation. Berzosertib was not removed or washed out during this period and remained in the medium. A cartoon of the experimental design is depicted in Supplementary Fig. 3G. We examined the plates with brightfield-microscopy to determine the number of positive wells. A well containing a colony with over 50 cells was considered positive for clonogenic cell growth. The ELDA software (http://bioinf.wehi.edu.au/software/elda/, [28]) was used to calculate clonogenicity and survival fractions. Calculation of irradiation doses required to reach 50% survival was done using Graphpad-Prism 7.3.

### Spheroid growth assay

The spheroid growth assay was carried out as described elsewhere [31, 32]. In brief, cells were seeded at a density of 50 cells per well in 96-well ultra-low attachment plates. After 48 h, when spheroids were formed, they were treated with three different doses of berzosertib (66.7%*IC20, 100%*IC20 and 150%*IC20) and irradiated 2 h later with 0, 2 or 4 Gy. After irradiation, spheroids were observed for 20–35 days. Photos were taken with brightfield microscopy (Leica DMi8, Leica, Germany). The LAS X Office was used for the evaluation of the spheroid-size. The largest width A of the spheroid and the perpendicular breadth B were measured. In approximation, the spheroids were considered as ellipsoids. Their volume was calculated with the formula

$$V=\,\frac{4}{3}\pi * A* B* \sqrt{A* B}$$

*V*: *Volume*, *A*: *Width*, *B*: *Breadth*

### Spheroid viability assay

In parallel to spheroid growth, spheroid viability was assessed at two timepoints by staining cells with Hoechst (alive) and propidium iodide (PI, dead) as described elsewhere [33]. Briefly, the dyes were added to the spheroids at a final concentration of 2.5 µg/mL for Hoechst and 20 µg/mL for PI. After 2 h of incubation, acquisition was made with fluorescence microscopy (Leica DMi8, Leica, Germany) with 10 ms (Hoechst) or 30 ms (PI) excitation times.

### Chorioallantoic membrane (CAM)-assay

The CAM assay was used as a proof-of-concept method to evaluate the radiosensitizing effect of berzosertib mimicking in vivo conditions and it was carried out as previously described [25, 26, 34]. In brief, chicken eggs were incubated in the environment of relative air humidity of 65% and a temperature of 37 °C, with automatic turning four times a day for 10 days before grafting to ensure proper embryo development. Therefore, DMG cells were pretreated 48 h prior to grafting with 100%*IC20 of berzosertib, irradiation (0, 2 or 4 Gy) or the combination of both. A small hole was made on the bottom of the egg to allow the CAM to be lowered and the eggshell was opened with a drill, disclosing the CAM of the egg on the day of grafting. The cells were harvested, and two million cells were mixed in 10 µL medium combined with 10 µL Corning Matrigel Growth Factor Reduced (GFR) Basement Membrane Matrix (Corning, USA) and put on the CAM above a large vessel. Tumors were harvested 7 days after grafting, and their diameter and weight were measured.

### Illustrative cartoon

The cartoon depicting the protocol of limiting dilution assay in Supplementary Fig. 3G was created by using https://BioRender.com.

### Statistical analysis

Statistical analysis was performed by using GraphPad Prism 7.3. Additional analyses were performed in Microsoft Excel. Synergy was assessed using SynergyFinder-Plus (https://synergyfinder.fimm.fi, [27]). Assuming a normal distribution, statistical significance was calculated either using an unpaired Student´s t-test or the one-way analysis of variance (ANOVA) with the following post-hoc Tukey test. 95% was defined as the confidence interval. The *p*-value of ≤0.05 was considered significant. Asterisks (*) indicate significance as follows: *p < 0.05, **p < 0.01, ***p < 0.001, ****p < 0.0001.

## Results

### High-throughput screening revealed enhanced antineoplastic effects of radiotherapy in combination with the ATR inhibitor berzosertib in DMG models in vitro

High-throughput drug screening using 687 compounds was performed on all seven DMG cell lines in conjunction with 4 Gy irradiation exposure at 0 and 24 h for a total of 8 Gy (Fig. 1A). Supervised analysis based on general sensitivity to compounds (Supplementary Fig. 1) was performed using the area under the curve (AUC).

To enable a structured comparison of drug responses, we first grouped the seven DMG cell lines into Sensitive and Resistant subgroups based on their AUC values under non-irradiated conditions (Supplementary Fig. 1A). This grouping was supported by variance-filtered principal component analysis (top 10% most variable compounds ranked by standard deviation), which resulted in a clear separation between the two subgroups (Supplementary Fig. 1B).

Based on this Sensitive/Resistant stratification, we then performed a supervised analysis of compound effects under irradiation. After filtering for significance (*p* < 0.05, fold change ± 1.4) and excluding compounds already significant at 0 Gy, six compounds met the selection criteria. Among these, berzosertib showed the strongest differential activity between Sensitive and Resistant cell lines and was therefore prioritized for mechanistic and functional validation (Fig. 1B, C). Consistent with these findings, berzosertib, an ATR inhibitor, enhanced the growth-inhibitory effects of ionizing radiation across all seven DMG cell lines (Fig. 1B). Notably, berzosertib demonstrated significantly greater activity in the Sensitive group of cell lines, which exhibited a lower AUC compared to the Resistant group (0.544 versus 0.801) (Fig. 1B, C). The top hits identified based on *p*-value were also found to be more active in the sensitive group and further included compounds such as rabusertib (checkpoint kinase inhibitor) and verteporfin (YAP inhibitor) (Fig. 1B). Additionally, the dose-response curves clearly showed that the DMG cell lines in the Sensitive group had a lower average IC50 values compared to the Resistant group (Fig. 1D). This data suggested that berzosertib could serve as a potential radiosensitizer candidate for the treatment of DMG.

Published data on genetic profiling of five DMG cell lines used in this study [8] revealed frequent mutations in one or multiple genes involved in the DNA damage response and cell cycle regulation, e.g., the *Tumor Protein p53* (*TP53*), *Ataxia Telangiectasia Mutated* (*ATM*), *Protein Phosphatase, Mg*^2+^*/Mn*^2+^ *Dependent 1D* (*PPM1D*), and *AT-Rich Interaction Domain 1* *A* (*ARID1A*) (Fig. 1E), which is reminiscent of data from DMG H3K27 altered patients [2, 9, 35]. Despite their frequent occurrence, mutations in DNA damage response and cell cycle regulation genes were not significantly associated with drug sensitivity differences among the cell lines (Supplementary Fig. 1C). To cover both phenotypic and molecular diversity of DMG H3K27-altered, we therefore selected SU-DIPG-17, SU-DIPG-24, and SU-DIPG-33 for the subsequent validation experiments. These three cell lines represented the most sensitive and most resistant cell lines of the drug screen and include the only HIST1H3B/C-K27M-mutant SU-DIPG-33 cell line (Supplementary Fig. 1C).

### Berzosertib exerts synergistic inhibitory effects with radiotherapy on proliferation of DMG cell lines

**Fig. 2:**
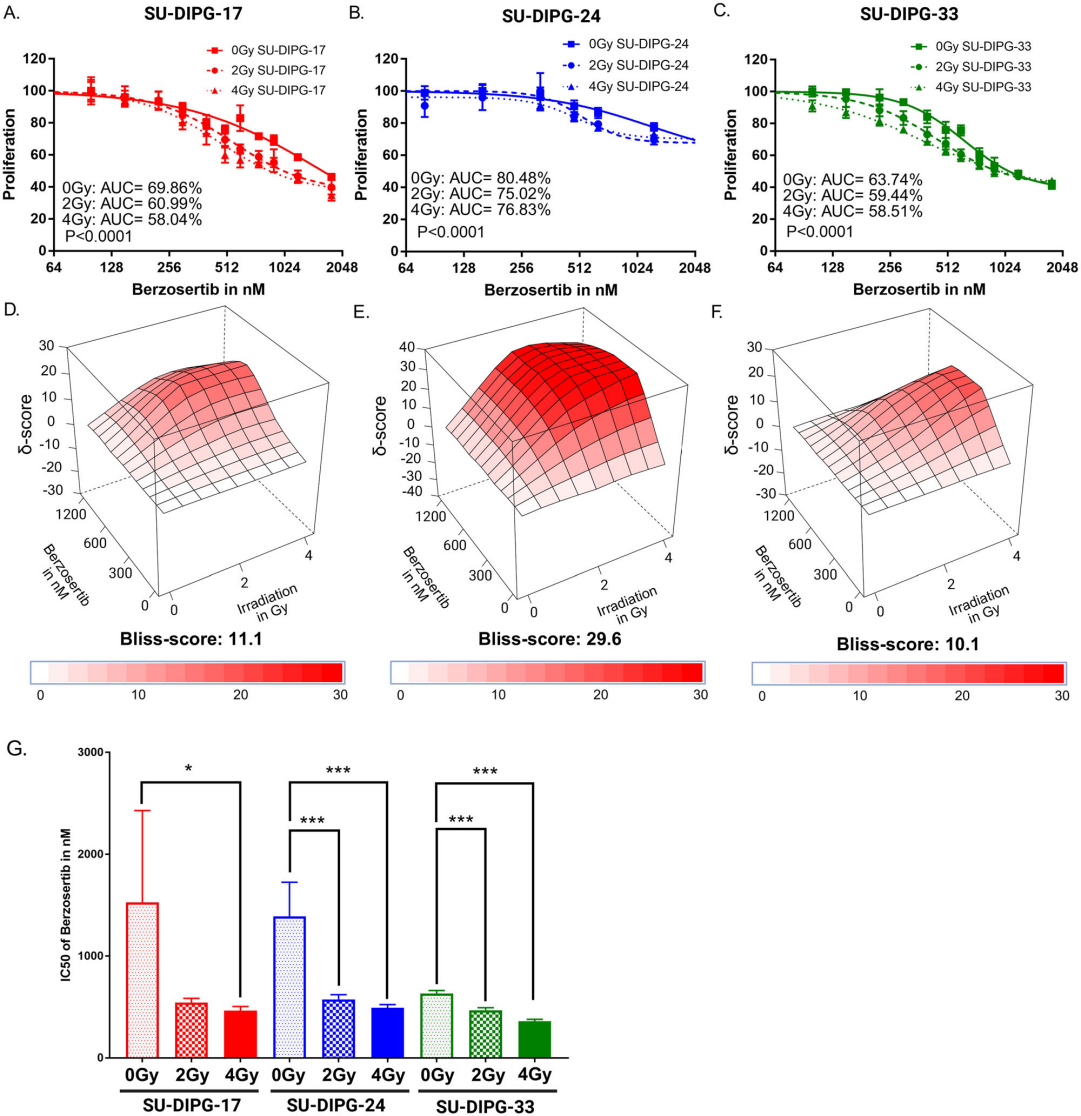
Berzosertib enhanced the antineoplastic effects of ionizing irradiation in DMG cell lines in short-term assays. Results from proliferation assays of DMG cell lines treated with 10-12 concentrations of berzosertib and 0, 2 or 4 Gy. 72 h after treatment proliferation was measured with CellTiter-Glo. Exemplary proliferation assays for the three tested cell lines SU-DIPG-17 (**A**) SU-DIPG-24 (**B**) and SU-DIPG-33 (**C**). Proliferation was normalized to control without berzosertib for 0, 2 or 4 Gy separately. IC20 and IC50 values were derived from nonlinear regression of dose-response curves as described in Materials and Methods. Bliss-Synergy score 3D plots for SU-DIPG-17 (**D**) SU-DIPG-24 (**E**) and SU-DIPG-33 (**F**). Red color/value above 10 indicates a high probability of synergism, whereas values below -10 indicate antagonism and values between -10 and 10 indicate an additive effect. Overall Bliss scores and color scales are depicted below each plot. **G** IC50 overview of DMG cell lines (SU-DIPG-17: 0 Gy: 1526 nM, 2 Gy: 542.6 nM, 4 Gy: 463,6 nM; SU-DIPG-24: 0 Gy: 1390 nM, 2 Gy: 575.2 nM, 4 Gy: 492.6 nM; SU-DIPG-33: 0 Gy: 631.8 nM, 2 Gy: 468.6 nM, 4 Gy: 359.7 nM). Error bars show standard deviation. Statistical analysis was performed using one-way ANOVA comparison and with following Tukey-test. ns not significant (*p* > 0.05), *p < 0.05, **p < 0.01, ***p < 0.001, ****p < 0.0001. Parentheses above bars indicate significance between compared groups.

As outlined above, our high-throughput drug screening identified the ATR inhibitor berzosertib as a leading radiosensitizer candidate in pediatric DMG cell lines. Next, we aimed to validate the suspected combined action of berzosertib and ionizing radiation and explore potential additive or synergistic effects. We first investigated the effects of berzosertib-treatment on radiation-induced growth inhibition of SU-DIPG-17, SU-DIPG-33 and SU-DIPG-24 cells in short-term proliferation assays (Fig. 2A–C). Therefore, we selected berzosertib-concentrations based on the data from our high-throughput screening and from the literature [36–39].

Berzosertib inhibited proliferation of DMG cells when given alone; however, drug treatment was significantly more effective in reducing the number of viable SU-DIPG-17 (Fig. 2A), SU-DIPG-24 (Fig. 2B), and SU-DIPG-33 (Fig. 2C) cells in combination with exposure to ionizing radiation with 2 Gy or 4 Gy. As expected from the more resistant behavior in the drug screen, SU-DIPG-24 cells displayed elevated AUC values after both, single-agent berzosertib and combination treatments, compared to SU-DIPG-17 and SU-DIPG-33 cells (Fig. 2A-C). Nevertheless, determination of the Bliss synergy score revealed potent synergistic effects in all three cell lines, as indicated by significantly reduced scores greater than ten when combining berzosertib (300–1250 nM) with 2 Gy or 4 Gy irradiation (Fig. 2D–F and Supplementary Fig. 2D–F). The IC50 was defined from the proliferation assays (Fig. 2A–C and Supplementary Fig. 2A–C) as the concentration producing 50% of the maximal inhibitory effect of berzosertib, and the IC20 as the concentration achieving 20% of the maximal inhibitory effect, based on the fitted dose-response curve under each irradiation condition (0, 2, and 4 Gy; see Materials and Methods). The IC20 in combination with 4 Gy irradiation was used as the reference concentration for all subsequent long-term experiments (Supplementary Fig. 2A–C). Importantly, combined treatment with 4 Gy irradiation significantly reduced the IC50 of berzosertib by 70.7% for SU-DIPG-17, 60.3% for SU-DIPG-24 and 42.3% for SU-DIPG-33 compared to drug treatment alone (Fig. 2G, *P* < 0.05, Supplementary Fig. 2G).

Collectively, these results indicated that berzosertib strongly synergizes with the growth inhibitory effect of ionizing radiation across all three DMG cell lines tested.

### Berzosertib treatment significantly enhances radiosensitivity of DMG cells in long-term assays

To investigate whether the berzosertib-induced increase in short-term growth inhibitory effects of ionizing radiation translates into reduced survival of clonogenic tumor cells in long-term assays, and thus radiosensitivity, we performed limiting dilution assays (LDA) in the three DMG cell lines. Therefore, we combined various radiation doses (0, 2, 4, and 8 Gy) with berzosertib concentrations representative of 33.3%, 66.7%, and 100% of the IC20 value. These concentrations are referred to as 33.3%*IC20, 66.7%*IC20 and 100%*IC20 in the subsequent sections. Berzosertib was always administered 2 h prior to irradiation and was kept on the cells for 14 days (Supplementary Fig. 3G).

**Fig. 3:**
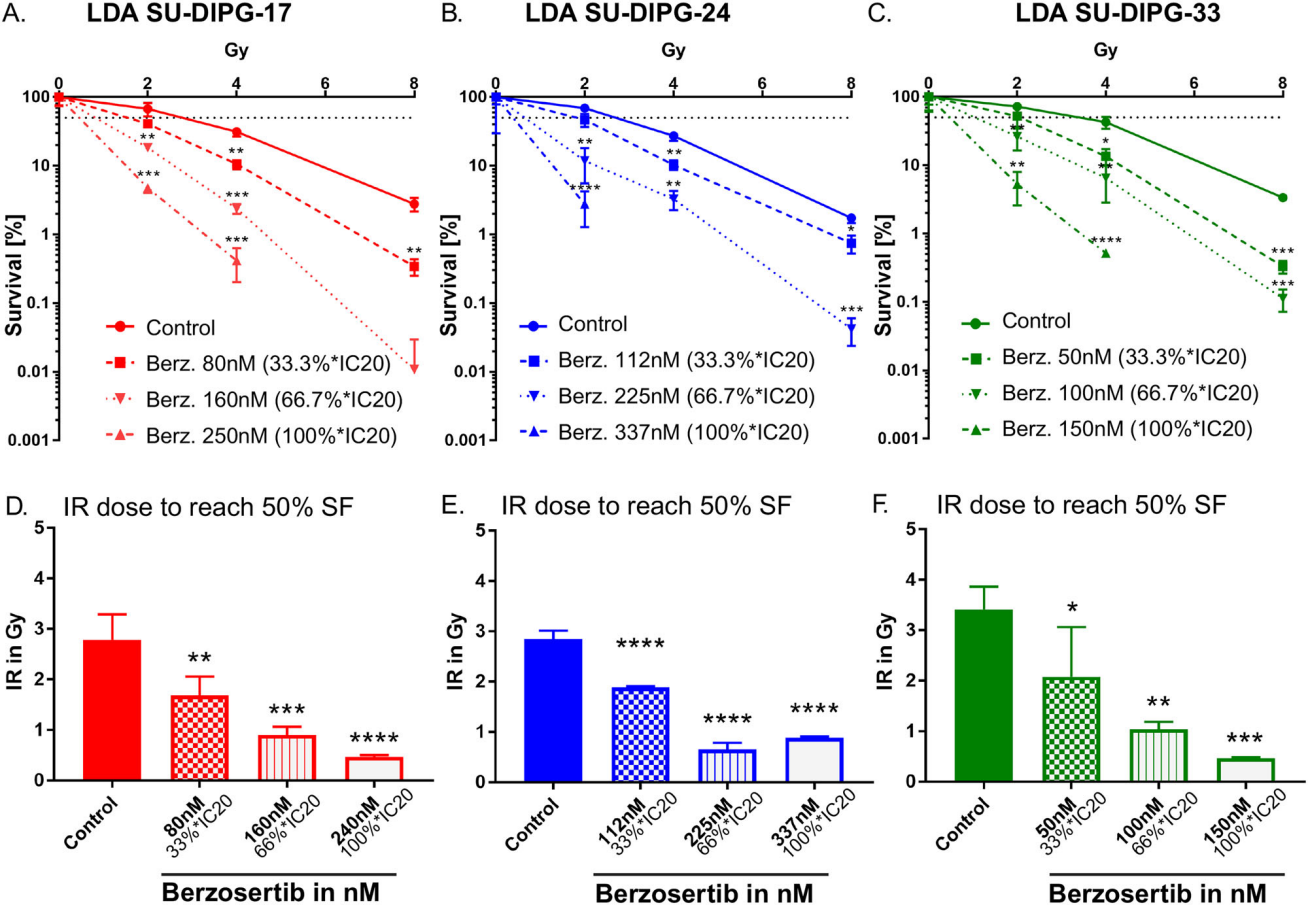
Berzosertib treatment enhanced radiosensitivity of DMG cell lines in long-term assays. Limiting-Dilution-Assay results for SU-DIPG-17 (**A**) SU-DIPG-24 (**B**) and SU-DIPG-33 (**C**) normalized to 0 Gy for each berzosertib concentration separately. Dotted line marks 50% survival. Statistical analysis was performed using paired t-Test for log-transformed probability values. No points displayed, when survival was 0% (100%*IC20 combined with 8 Gy in three cell lines and 100%*IC20 with 4 Gy in SU-DIPG-24). Calculated irradiation doses for reaching a survival fraction of 50% for SU-DIPG-17 (**D**) SU-DIPG-24 (**E**) and SU-DIPG-33 (**F**) depending on applied berzosertib concentration (33.3%*IC20, 66.7%*IC20, 100%*IC20). Values have been calculated with linear-quadratic-model curves fitted to each triplicate. Error bars show standard deviation. Statistical analysis was performed using one-way ANOVA and with following Tukey-test. ns not significant (*p* > 0.05), *p < 0.05, **p < 0.01, ***p < 0.001, ****p < 0.0001. Asterisks above bars indicate comparison with respective control.

Strikingly, our experiments revealed a significant decrease in the survival fraction when combining clinically relevant doses of 2 or 4 Gy irradiation with a berzosertib concentration of 66.7%*IC20 or 33.3%*IC20, respectively, compared to irradiation alone (Fig. 3A–C).

The radiation dose required to reduce survival fractions of the three DMG cell lines by 50% (SF50) amounted to 2.8 ± 0.5 Gy (SU-DIPG-17) to 3.2 ± 0.5 Gy (SU-DIPG-33) in single treatments. Berzosertib treatment significantly lowered the SF50 in all tested cell lines (Fig. 3D–F). While the effects became already visible at the lowest berzosertib concentration (33.3%*IC20), the highest berzosertib concentration (100%*IC20), reduced the SF50 dose below 1 Gy.

To directly compare the effect of single treatments vs combined treatments we additionally calculated survival curves normalized to the plating efficiency of the untreated controls (0 Gy; 0 nM berzosertib). Neither 8 Gy irradiation alone nor the highest berzosertib concentration (100%*IC20) alone reduced DMG cell survival below 1% of untreated controls. In contrast, combined treatment with berzosertib and ionizing radiation reduced cell survival below 0.1% of untreated controls in all three cell lines (Supplementary Fig. 3A–C). For comparison, we also evaluated the clinically used DNA-alkylating agent temozolomide by using concentrations representative of 33.3%, 66.7%, and 100% of the IC20 value in the same LDA setup. At the concentrations tested, temozolomide did not enhance the effects of 2 or 4 Gy irradiation in any of the cell lines (Supplementary Fig. 3D–F), underscoring the distinct radiosensitizing capacity of berzosertib.

Together, these findings demonstrate potent and consistent radiosensitizing effects of berzosertib in DMG cells in long-term clonogenic assays.

### Combining berzosertib treatment and irradiation suppressed the spheroid growth of DMG cells

**Fig. 4:**
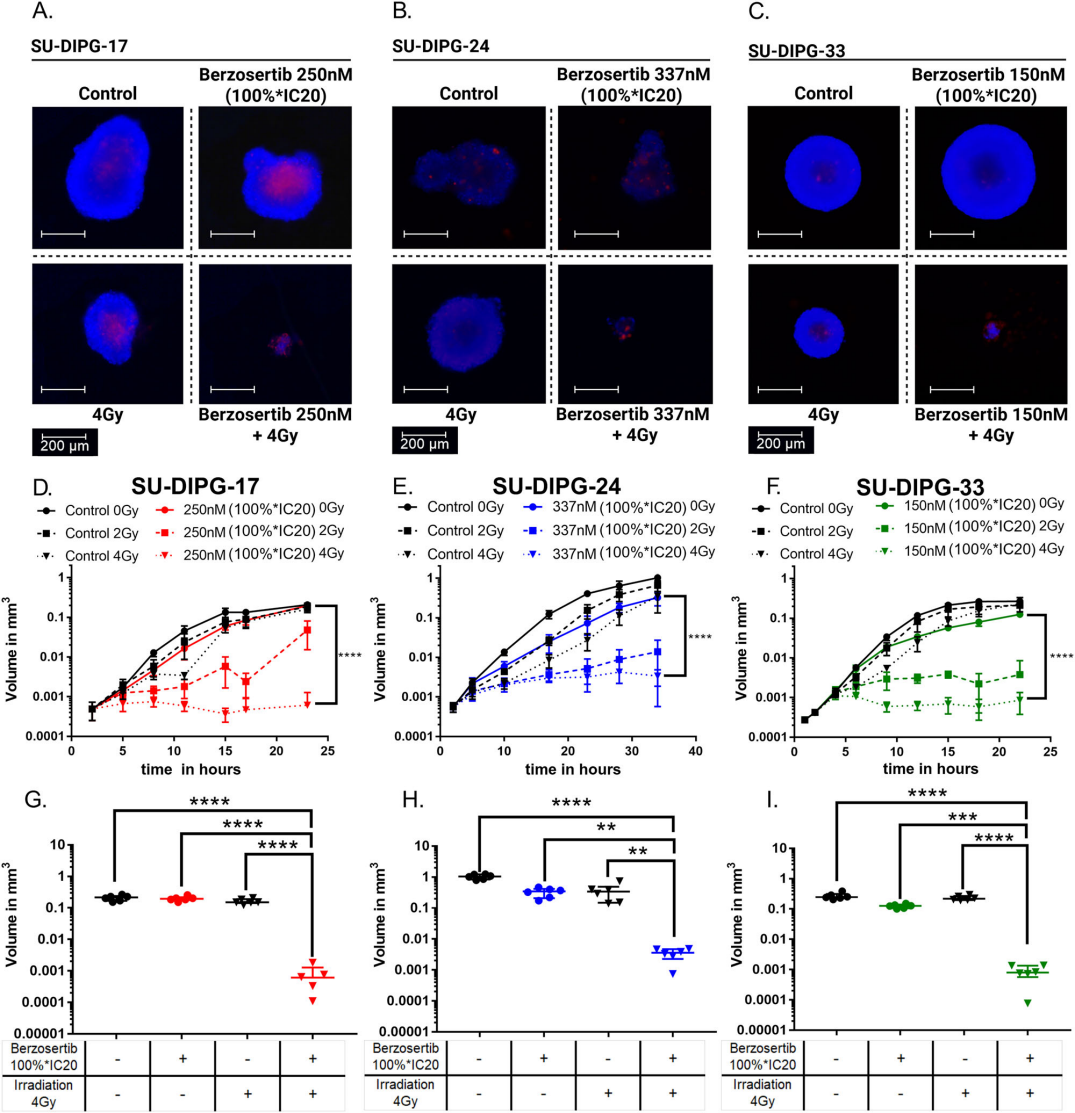
Berzosertib enhanced the radiation-induced growth delay of DMG-spheroids. For each spheroid 50 cells/ well have been plated in a ULA-96-well-plate. After 48 h, the cells were treated with berzosertib (as indicated) and Irradiation (4 Gy). After treatment the spheroids were observed for 20-35 days with brightfield microscopy and viability staining. Representative images of SU-DIPG-17 (**A**), SU-DIPG-24 (**B**) and SU-DIPG-33 (**C**) spheroids stained with Hoechst (viable) and Propidium-Iodide (dead) to assess viability at 10 (SU-DIPG-17, SU-DIPG-33) or 20 (Su-DIPG-24) days after application of indicated treatments. Spheroid volume growth curves over time for SU-DIPG-17 (**D**) SU-DIPG-24 (**E**) and SU-DIPG-33 (**F**) treated with 0, 2 or 4 Gy and 100%*IC20 of berzosertib. Significance tested with one-way ANOVA between 0 Gy and 100%*IC20 berzosertib as well as 4 Gy and 100%*IC20 berzosertib at the endpoint, *p* < 0.001. Error bars show standard deviation. Spheroid volume at the end of the measurement for SU-DIPG-17 (**G**) SU-DIPG-24 (**H**) and SU-DIPG-33 (**I**). Each dot represents a single spheroid. Significance tests conducted between combinational treatment and irradiation (4 Gy), drug treatment alone (100*IC20 of berzozertib) or control with one-way ANOVA showing *p* < 0.001 for all three cell lines. Error bars show standard deviation. ns not significant (*p* > 0.05), *p < 0.05, **p < 0.01, ***p < 0.001, ****p < 0.0001. Parentheses above bars indicate significance between compared groups.

Next, we utilized an additional and more physiologically relevant 3D spheroid model to further validate the radiosensitizing effects of berzosertib. We captured images of the spheroids at multiple time points (at least six) throughout the observation period to monitor alterations in spheroid growth. In addition, we performed live-dead staining with propidium iodide followed by fluorescence microscopy to assess treatment-induced cell death after the different treatments (Fig. 4A–C). This enabled us to identify distinct growth kinetics, including delayed recovery following initial growth arrest or late volume reductions after an initial period of spheroid growth. SU-DIPG-17 and SU-DIPG-33 spheroids exhibited rapid initial growth and reached a plateau by day 24 post-treatment (endpoint) (Fig. 4D, F). Instead, the SU-DIPG-24 spheroids grew more slowly, requiring an extended observation for up to 35 days (Fig. 4E).

Investigation of time-dependent treatment responses revealed that single treatments with 2 or 4 Gy irradiation or berzosertib concentrations of 100%*IC20 only slightly delayed spheroid growth. In contrast, prominent volume reductions were observed in all three cell lines when combining 100%*IC20 berzosertib and 4 Gy irradiation (Fig. 4D–F). Herein, SU-DIPG-24 required a higher absolute berzosertib concentration and displayed a delayed response to combined treatment compared to SU-DIPG-33 and SU-DIPG-17 spheroids (Fig. 4D–F). Direct comparison of spheroid volume at the end of the experiment demonstrated that the combination of 4 Gy irradiation and 100%*IC20 berzosertib significantly suppressed long-term spheroid growth compared to the single treatments in all three cell lines (Fig. 4G–I). Live-dead staining with propidium iodide- and subsequent fluorescence microscopy confirmed increased cell death in spheroids treated with berzosertib and irradiation, as exemplarily depicted in Fig. 4A–C.

Similar results were obtained when observing dose- and concentration-dependent combination effects (Supplementary Fig. 4A-C): When treating DMG spheroids with various berzosertib concentrations (66,7%*IC20, 100%*IC20, or 150%*IC20) and irradiation (0, 2, or 4 Gy) we observed significant volume reductions with suggested clinical relevance (volume decrease by more than x 0.001) when combining 2 Gy irradiation and 100%*IC20 berzosertib (SU-DIPG-17; SU-DIPG-33) or 4 Gy irradiation and 150%*IC20 (SU-DIPG-24), respectively (Supplementary Fig. 4A–C).

Altogether, the findings in this section demonstrate that potent, concentration-dependent radiosensitizing effects of berzosertib in combination with clinically relevant irradiation doses also occur in a physiologically relevant 3D spheroid model.

### In ovo experiments corroborated synergistic growth inhibition of DMG cells when combining berzosertib treatment with a single dose of irradiation

**Fig. 5:**
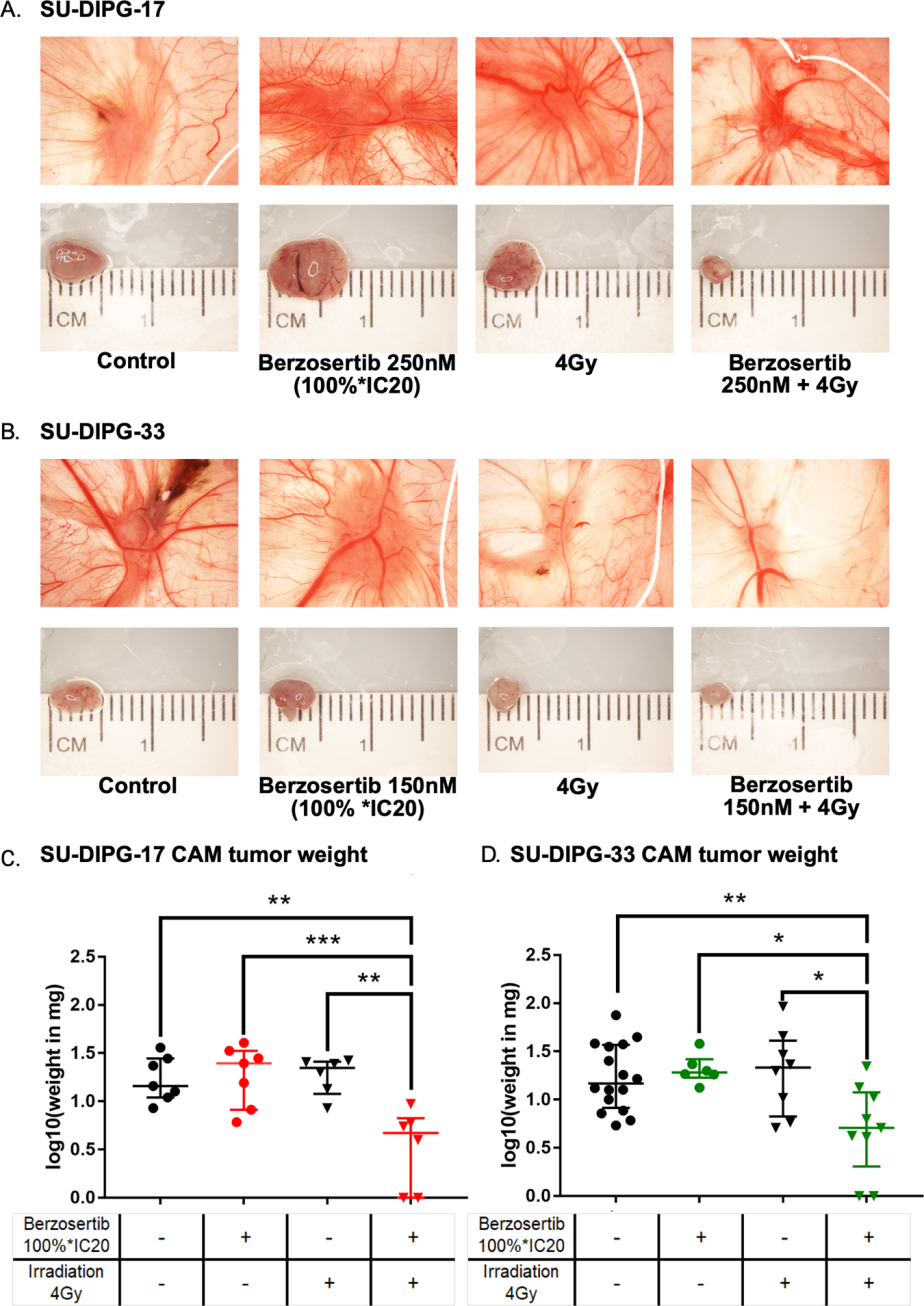
Pre-treatment with combined berzosertib and irradiation significantly decreased tumor growth *in ovo*. SU-DIPG-17 and SU-DIPG-33 cells were treated with 100%* IC20 berzosertib, irradiated 2 h later with 0, 2 or 4 Gy, seeded on the CAM-membrane of eggs. Membrane and tumors were collected 7 days after from the CAM. Representative images of tumors harvested on day 7 from the CAM of the cell lines SU-DIPG-17 (**A**) and SU-DIPG-33 (**B**). Overview of the weight of the harvested CAM tumors after separation from the CAM of the cell lines SU-DIPG-17 (**C**) and SU-DIPG-33 (**D**). One-way ANOVA was used for comparisons between treatments. ns not significant (*p* > 0.05), *p < 0.05, **p < 0.01, ***p < 0.001, ****p < 0.0001. Parentheses above bars indicate significance between compared groups.

Finally, we used the CAM model to validate the combination effect of berzosertib treatment and irradiation on the growth of SU-DIPG 17 (Fig. 5A) and SU-DIPG-33 (Fig. 5B) tumors in proof-of-principle experiments *in ovo*, mimicking more complex in vivo tumor conditions. Cells were pre-treated with berzosertib at 100*IC20 concentrations, subjected to irradiation with a dose of 4 Gy, and allowed to recover for 48 h prior to seeding on the CAM. Consistent with previous experiments, the 100%*IC20 concentrations were employed for SU-DIPG-17 (250 nM) and SU-DIPG-33 cells (150 nM).

Single-agent treatments with either berzosertib or irradiation alone did not significantly alter tumor growth in the CAM model (Fig. 5C, D). However, combining pre-treatment with the respective berzosertib 100%*IC20-concentration with a 4 Gy irradiation resulted in significantly reduced tumor weight compared to controls (SU-DIPG-17: *p* = 0.0016 and SU-DIPG-33: *p* = 0.0078; Fig. 5C, D, respectively). These findings demonstrated that berzosertib markedly potentiates the antitumoral effects of irradiation, effectively suppressing tumor formation of DMG cells *in ovo*. Furthermore, these results validated the radiosensitization effects previously observed in limiting dilution and spheroid assays, highlighting the therapeutic potential of berzosertib when combined with clinically relevant radiation doses.

In summary, our data validated berzosertib as a potent radiosensitizer in vitro (2D and 3D models) as well as *in ovo*, supporting its potential clinical application for enhancing the therapeutic impact of radiotherapy in DMG patients.

## Discussion

Up to now, radiotherapy remains the mainstay treatment approach that provides a modest survival benefit for patients suffering from diffuse midline glioma H3K27M-altered (DMG). Though radiotherapy typically delays tumor progression by approximately three to six months, dismal survival rates of less than 10% beyond two years highlight an urgent clinical need for the identification of compounds that enhance the efficacy of radiotherapy. High-throughput drug screening of molecularly representative DMG cell lines in combination with ionizing radiation allowed us to identify compounds that increased the effects of ionizing radiation in short-term assays, including the ATR inhibitor berzosertib. In subsequent analyses we validated promising antineoplastic and radiosensitizing effects of berzosertib using state-of-the art short- and long-term assays (2D), as well as the clinically more relevant spheroid growth assay (3D). Importantly, proof-of-principle experiment in a first in vivo setting using the CAM model corroborated the potent combinatorial growth inhibitory effects of berzosertib with radiotherapy. Our data provide a rationale for exploring the use of this novel treatment concept in DMG patients.

Despite heterogeneity in the extent of berzosertib-mediated combination effects with radiotherapy among the tested DMG cell lines, we observed synergy between berzosertib and ionizing radiation in all three DMG cell lines. Furthermore, berzosertib demonstrated more pronounced combination effects in SU-DIPG33 and SU-DIPG17 compared to SU-DIPG24 cells, as indicated by lower IC20 concentrations in combination with 4 Gy.

Additional exploratory long-term LDA assays identified berzosertib as the most promising radiosensitizer derived from our HTS experiments compared to other drugs. Consistent with the LDA data, temozolomide showed no measurable radiosensitizing effect in the LDA assay at the concentrations tested, underscoring the specificity of berzosertib-mediated enhancement at the concentrations used. The pronounced radiosensitizing effects of berzosertib at 2 Gy and 4 Gy are of particular interest for clinical radiotherapy since 2 Gy is frequently used in conventional fractionated radiotherapy, whereas 4 Gy represents a clinically relevant dose for hypofractionation treatment regimes.

Importantly, we were able to validate synergistic effects between berzosertib and radiotherapy in two more clinically relevant experimental settings using a 3D spheroid growth assay as well as proof-of-principle in vivo experiments in the CAM model. Both assays highlighted time, drug concentration and radiation dose-dependent effects of treatments combining berzosertib and ionizing radiation and will allow us to derive optimal treatment conditions for experiments validating fractionated irradiation in further preclinical in vitro studies, and future proof-of-concept experiments in vivo in an orthotopic murine model.

The synergy of berzozertib in combination with radiotherapy observed in DMG cell lines aligns with preclinical and clinical findings in other tumor types: In fact, ATR inhibition has been found to enhance the effects of both, radiotherapy and chemotherapy, in the nanomolar range (50–1000 nM), particularly in cancers with defects in DNA damage response pathways [36, 40, 41].

Even more important, the concentrations used in the present study have been described to be achieved two hours after drug application in brain tissue and plasma in vivo [36] and can thus be considered as clinically relevant.

Berzosertib belongs to the drug class termed DNA damage response (DDR) inhibitors that had initially been developed to exploit synthetic lethality with DNA repair defects specific to cancer cells. A prime example is the seminal discovery by Alan Ashworth and colleagues about the synthetic lethality of PARP-1 inhibitors in BRCA1/2-deficient tumors [42]. In this context, radiotherapy is expected to broaden the application of DDR inhibitors to cancers without defined DNA repair defects or other mutations due to its localized induction of DNA damage to tumors when using highly conformal radiotherapy techniques.

The kinase ATR regulates cell cycle checkpoints, replication fork stability and DNA repair through a phosphorylation cascade including the checkpoint kinase Chk1. ATR is mainly activated in response to replication-associated damage to promote cell survival in cells with replication stress and DNA damage [43]. Mechanistically, ATR inhibition is thought to abrogate the G2/M cell cycle checkpoint, increase DNA damage induction, and enhance DNA damage persistence, thereby increasing tumor cell vulnerability to radiation-induced lethality [44, 45]. Interestingly, molecular alterations causing enhanced replication stress or compromising G1 cell cycle checkpoints control, e.g. mutations in p53 or in the ATM-p53 pathway, confer vulnerability to ATR inhibitors [40, 41, 46], which may be further increased in combination with genotoxic chemo-/radiotherapies [40, 43, 46]. A recent preclinical study further described genotype-directed synthetic growth suppression of ATR inhibition together with radiotherapy in cancer cells with ATM defects in triple-negative breast and colorectal cancer murine models [41].

Of note, publicly available datasets revealed frequent mutations in genes involved in the DNA damage response and cell cycle regulation such as TP53, ATM, PPM1D and ARID1A among DMG cell lines, including the cell lines used in the present study (Fig. 1E). It is thus highly likely that these genetic alterations may contribute to the pronounced berzosertib-mediated radiosensitization in the DMG cell lines. Interestingly, the second most effective compound identified in our high-throughput screening was the checkpoint kinase 1 inhibitor rabusertib. Rabusertib acts downstream of berzosertib but in the same pathway, thereby further supporting the suggested potential mechanistic basis of our findings outlined above. In line with these observations, our high-throughput screen also identified ceralasertib, a structurally distinct and highly selective ATR inhibitor, as an active compound in several DMG cell lines. This independent identification of two chemically different ATR inhibitors supports the possibility that the observed enhancement of radiation responses may involve ATR pathway modulation. However, future investigations are necessary to fully understand the extent to which ATR pathway engagement specifically contributes to radiosensitization and to explore its underlying mechanisms in different DMG subtypes. A deeper understanding of these mechanisms may also help to identify molecular features associated with differential treatment responses across DMG cell lines.

Looking for additional potential correlations to mutational signatures we observed, several alterations present in SU-DIPG-17 and SU-DIPG-33, including amplifications in *HRAS, MYC, PI3KCA*, and defects in *BRCA1/2, PARP1/2, RAD51*, are known to increase replication stress or impair high-fidelity DNA-damage repair [37, 43, 47–49]. Such backgrounds have previously been associated with enhanced sensitivity to ATR inhibition and to genotoxic therapies [37, 43, 47–49]. Together with the short doubling times reported for these cell lines [8], these features may facilitate accelerated mitotic catastrophe when ATR is inhibited, thereby enhancing combination effects in short-term assays. SU-DIPG-19 represents a distinct scenario, as it has no reported p53 alterations but carries a loss of *ATM*, a recurrent alteration in ~20% of DMG H3K27-altered lines. *ATM* loss is known to create synthetic lethality with ATR inhibition [45, 50–52], providing a plausible explanation for the pronounced synergy observed in this line, even in the absence of extensive additional oncogenic lesions.

Overall, the three sensitive lines share features expected to elevate replicative stress or compromise checkpoint control, both of which may potentiate ATR-inhibitor-mediated radiosensitization. However, since SU-DIPG-24 and SU-DIPG-25 also harbor amplifications of *KRAS* or *MYC* without showing strong synergy in the short-term screen, no single alteration appears determinative. So far, there is no clear correlation between mutational signatures and sensitivity to berzosertib in combination with ionizing radiation. Instead, our findings support the concept that a combination of replication-stress-associated changes, DNA-repair defects, and proliferation kinetics may modulate short-term sensitivity to berzosertib in combination with radiation. Further mechanistic work is ongoing to provide insight into the underlying mechanisms and thereby to identify biomarkers of response in DMG.

Several recent studies have highlighted the central importance of p53 and the ATM/ATR checkpoint pathways in shaping the radiation response of DMG H3K27-altered tumors. In clinical and preclinical studies, p53 mutation has consistently been associated with poor prognosis and reduced responsiveness to radiotherapy [53–55]. These findings correspond to experimental models showing a pronounced radioresistance of p53-mutant DMG [54, 55]. To overcome this therapy resistance, several groups have explored inhibition of DNA-damage checkpoints, including the G1/S (p53-ATM) and G2/M (ATR-CHK1-WEE1) axes [53, 55, 56]. In a landmark study, Mangoli et al. demonstrated enhanced radiosensitivity in ATM-null / p53-mutant DMG, and improved survival upon combination of radiotherapy with the ATM inhibitor AZD1390, whereas ATM loss alone did not sensitize tumors with wild-type p53 or p53 allelic variants [54, 55, 57]. A separate study in mouse models reported no radiosensitization by ATM inhibition in DMG harboring wild-type p53 but mutant PTEN [58], highlighting the genetic context dependency of the pathway.

Beyond ATM, inhibition of ATR has also emerged as a promising strategy. Importantly, resistance of p53-mutant DMG to the topoisomerase-I inhibitor SN38 was reversed by ATR inhibition with AZ20 [56], consistent with the established role of ATR in managing replication stress induced by DNA single-strand and double-strand break-forming agents [59]. Furthermore, downstream components of the pathway like CHK1 [53] and WEE1 [57] have been identified as radiosensitization targets, although the WEE1 inhibitor adavosertib failed to improve survival in a first Phase I trial [21].

Together, these findings illustrate that multiple DNA-damage-response components modulate DMG sensitivity to radiation and DNA-damaging agents [53–56, 58, 59]. Herein, p53 dysfunction is both a driver of radioresistance and, at the same time, a therapeutic vulnerability, as tumors with impaired checkpoint control appear particularly susceptible to ATR pathway inhibition, which is consistent with the radiosensitizing effects observed in our study. However, the underlying mechanisms warrant further systematic investigations as they may allow discrimination between molecular DMG subgroups with distinct sensitivity.

Clinical studies have already demonstrated the clinical benefit of DDR inhibitors in combination with radiotherapy, e.g., the Wee1-inhibitor adavosertib in pancreatic cancer [60] or the PARP-inhibitor olaparib in breast cancer [61]. Further clinical studies observed a prognostic benefit of the ATR inhibitor berzosertib in combination with chemotherapy in small-cell lung cancer and ovarian cancer patients [37, 62], though berzosertib was also being tested in combination with radiotherapy in other difficult-to-treat malignancies [63, 64].

So far, investigations regarding radiosensitizing agents in pediatric DMG are still rare. We speculate that DMG with frequent mutations in TP53 and the ATM-p53 pathway [8] will be exquisitely sensitive to the lethal effects of ATR inhibition in combination with radiotherapy, providing a rationale for biomarker-driven clinical development. Herein, the ATR inhibitor gartisertib showed 4-fold greater potency than berzosertib in glioblastoma models, overcoming temozolomide resistance and enhancing radiation-induced cell death in DDR-mutant cell lines [65]. Furthermore, the availability of ATR inhibitors or other compounds acting in the ATR pathway with sufficient blood-brain barrier penetrance will be instrumental for the clinical success of such a treatment. Though berzosertib reached effective concentration in the brain of mice two hours after drug application, the drug was rapidly removed from the cells by MDR-proteins [36]. Notably, ceralasertib and AD1058, new promising selective ATR inhibitors, have been reported to penetrate the brain tissue [66, 67].

Taken together, we demonstrate that the ATR inhibitor berzosertib acts as a potent radiosensitizer for DMG in preclinical experiments at clinically relevant drug concentrations. Our comprehensive screening approach integrating drug sensitivity profiling with irradiation indicates novel pharmacological vulnerabilities and suggests rational opportunities to improve the efficacy of radiotherapy in a disease with limited treatment options and a fatal prognosis. The pronounced synergistic effects highlight the potential of ATR inhibition as a precision approach to improve the outcome of radiotherapy in DMG and the high medical need for identifying molecular biomarkers to guide patient selection for future clinical application. Though blood-brain-barrier penetrance of ATR inhibitors remains a challenge, the pronounced synergistic effects of ATR inhibition and radiotherapy observed in this study provide a rationale for therapeutic strategies to either intensify the benefit of radiotherapy by combined treatment or to de-escalate radiation doses to reduce adverse effects. Given the high clinical need for improving DMG radiotherapy, these findings warrant further validation in vivo and in clinical trials, alongside the development of compounds or novel delivery methods to improve brain availability of DDR inhibitors.

## Supplementary information

Supplementary Material

## Data Availability

This study did not use any unpublished custom code, software, or algorithm. Any additional information required to reanalyze the data reported in this paper is available from the lead contact upon request.

## Code availability

This study did not use any unpublished custom code, software, or algorithm. Any additional information required to reanalyze the data reported in this paper is available from the lead contact upon request.
