# Supplementary material for: Berzosertib enhances the sensitivity of pediatric diffuse midline glioma H3K27-altered cells to radiotherapy
Source: Cell Death Dis. 2026 Mar 20;17(1):331. doi: 10.1038/s41419-026-08567-7 (PMC13039724; doi:10.1038/s41419-026-08567-7)
Supplement: Supplementary file 1 — Supplementary Material [file 41419_2026_8567_MOESM1_ESM.docx]

Supplementary Material

**Berzosertib enhances sensitivity of pediatric diffuse midline glioma H3K27 altered cells to radiotherapy**

Nikita Gorainow^1^, Felix Sander^2^, Daniel Picard^2,3,4^, Marvin Christopher Frölich^1^ , Katharina Eul^1^, Sarah Etemadi Afshar^1^, Julia Asche^1^, Michelle Monje^5^, Eric Raabe^6^, Jasmin Bartl^2^, Arndt Borkhardt^2^, Guido Reifenberger^3^, Nicole Dünker^7^, Maike Busch^7^, David Pauck^3^, Nan Qin^8,9^, Johann Matschke^1,10*#^, Marc Remke^2,3,4#^, Verena Jendrossek^1,10,11*#^

**1** Institute of Cell Biology (Cancer Research), University of Duisburg-Essen, University Hospital Essen**,** Essen Germany

**2** Department of Pediatric Oncology, Hematology, and Clinical Immunology, Medical Faculty, University Hospital Düsseldorf, Düsseldorf, Germany

**3** Institute of Neuropathology, Heinrich Heine University Düsseldorf, Medical Faculty, and University Hospital Düsseldorf, Düsseldorf, Germany

**4** Department of Pediatric Hematology and Oncology, University Medical Center of Saarland, Saarland University, Homburg (Saar), Germany

**5** Department of Neurology and Neurological Sciences, Stanford University, Stanford, CA, USA

**6** Department of Oncology, Johns Hopkins University, Baltimore, Maryland, USA

**7** Center for Translational Neuro- and Behavioral Sciences, Institute of Anatomy II, Department of Neuroanatomy, Medical Faculty, University of Duisburg-Essen, 45147 Essen, Germany.

**8** Department of Hematology, Heinrich Heine University Düsseldorf, Medical Faculty, Düsseldorf, Germany

**9** Spatial & Functional Screening Core Facility, Medical Faculty, Heinrich Heine University, Düsseldorf, Germany

**10** German Cancer Consortium (DKTK) partner site Essen a partnership between DKFZ and University Hospital Essen, Essen, Germany.

**11** West German Comprehensive Cancer Center Essen (CCC-WTZ)

**^#^** Equal contributions

***Corresponding authors:**

Prof. Dr. Verena Jendrossek, Institute of Cell Biology (Cancer Research), University of Duisburg Essen, University Hospital Essen, Virchowstrasse 173, 45147 Essen, Germany; Phone:+49-201-723 3380; e-mail: verena.jendrossek@uk-essen.de

PD Dr. Johann Matschke, Institute of Cell Biology (Cancer Research), University of Duisburg Essen, University Hospital Essen, Virchowstrasse 173, 45147 Essen, Germany; Phone:+49-201-723 4234; e-mail: johann.matschke@uk-essen.de

**Supplementary Figure 1. (Related to Figure 1)**


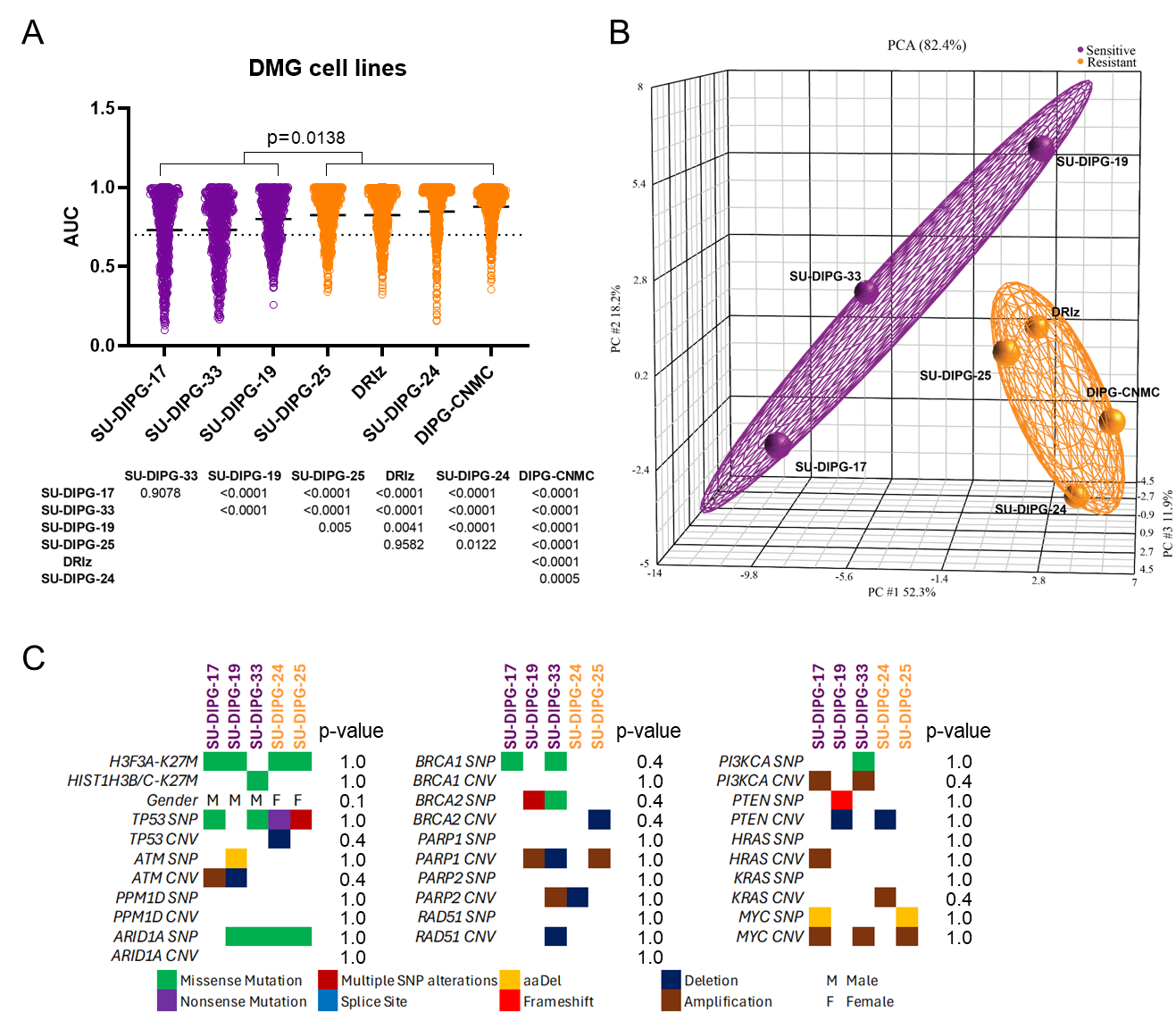
***Supplementary Figure 1. High-throughput screening of non-irradiated DMG cell lines suggests two groups:****(A) Area under the curve values for 687 compounds are displayed along with the average AUC (black bar) and ordered from lowest to highest AUC. The three samples with the lowest AUC are considered Sensitive (purple) and the other cell lines are considered Resistant (orange). Average AUC was used to calculate a p-value between groups (p=0.0138) and p value between all samples is provided below the graph. (B) 3D principal component analysis of the screening results with drug treamtant alone showing all seven DMG cell lines colored by their grouping (Sensitive vs Resistant) and using 10% of the drugs based on standard deviation. Ellipsoids show loose grouping of sensitive cell lines, whereas resistant lines form a smaller cluster. (C) Oncoprint of DMG cell lines showing key mutations in the characteristic histone genes and genes involved with DNA damage. Cell lines are highlighted according to their respective groups (Sensitive – purple, Resistant – orange). p-value denotes result of Fischer’s exact test.*

**Supplementary Figure 2. Berzosertib Enhances the short-term Cytotoxic Effect of Ionizing Irradiation in Diffuse-Midline-Glioma H3K27-altered (Related to Figure 2)**


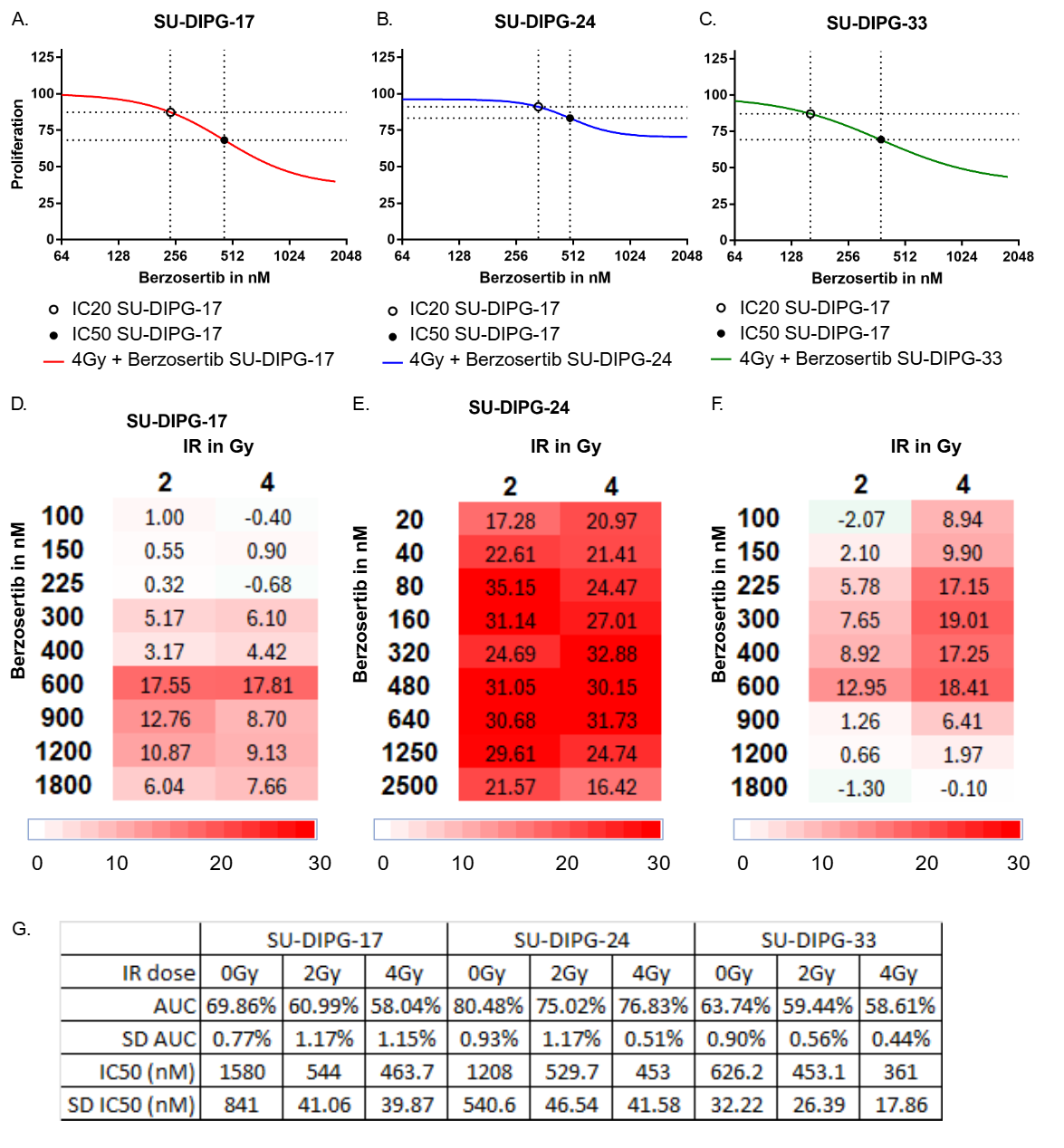


***Supplementary Figure 2.*** ***Berzosertib Enhances the short-term Cytotoxic Effect of Ionizing Irradiation in Diffuse-Midline-Glioma H3K27-altered:*** *Proliferation assay curves of the cell-lines SU-DIPG-17 (A), SU-DIPG-24 (B) and SU-DIPG-33 (C) treated with 4 Gy and varying concentrations of berzosertib. CellTiter-Glow was used for measuring proliferation. Proliferation was normalized to 4 Gy control without berzosertib for all three displayed curves. For all three cell-lines IC50 and the IC20 were determined as the berzosertib concentrations at which 50% and 20% of the maximal inhibitory effect have been achieved. The calculation was done using GraphPad Prism 7.0. Bliss-Synergy-score tables for SU-DIPG-17 (D), SU-DIPG-24 (E) and SU-DIPG-33 (F). Overall Bliss scores and color scales are depicted below each plot (D-F). Values below -10 indicate an antagonistism of the treatments. Values between -10 and 10 indicate an additive effect. Values above 10 indicate a synergy of the treatments. (G) Table showing primary read-outs of proliferation assays for SU-DIPG-17, SU-DIPG-24 and SU-DIPG-33 for the three irradiation doses 0, 2 and 4 Gy: Area-under-the-Curve (AUC), Standard-Deviation (SD) of AUC, IC50 and SD of IC50.*

**Supplementary Figure 3. Berzosertib Enhances the long-term Cytotoxic Effect of Ionizing Irradiation in Diffuse Midline Glioma H3K27-altered (Related to Figure 3)**


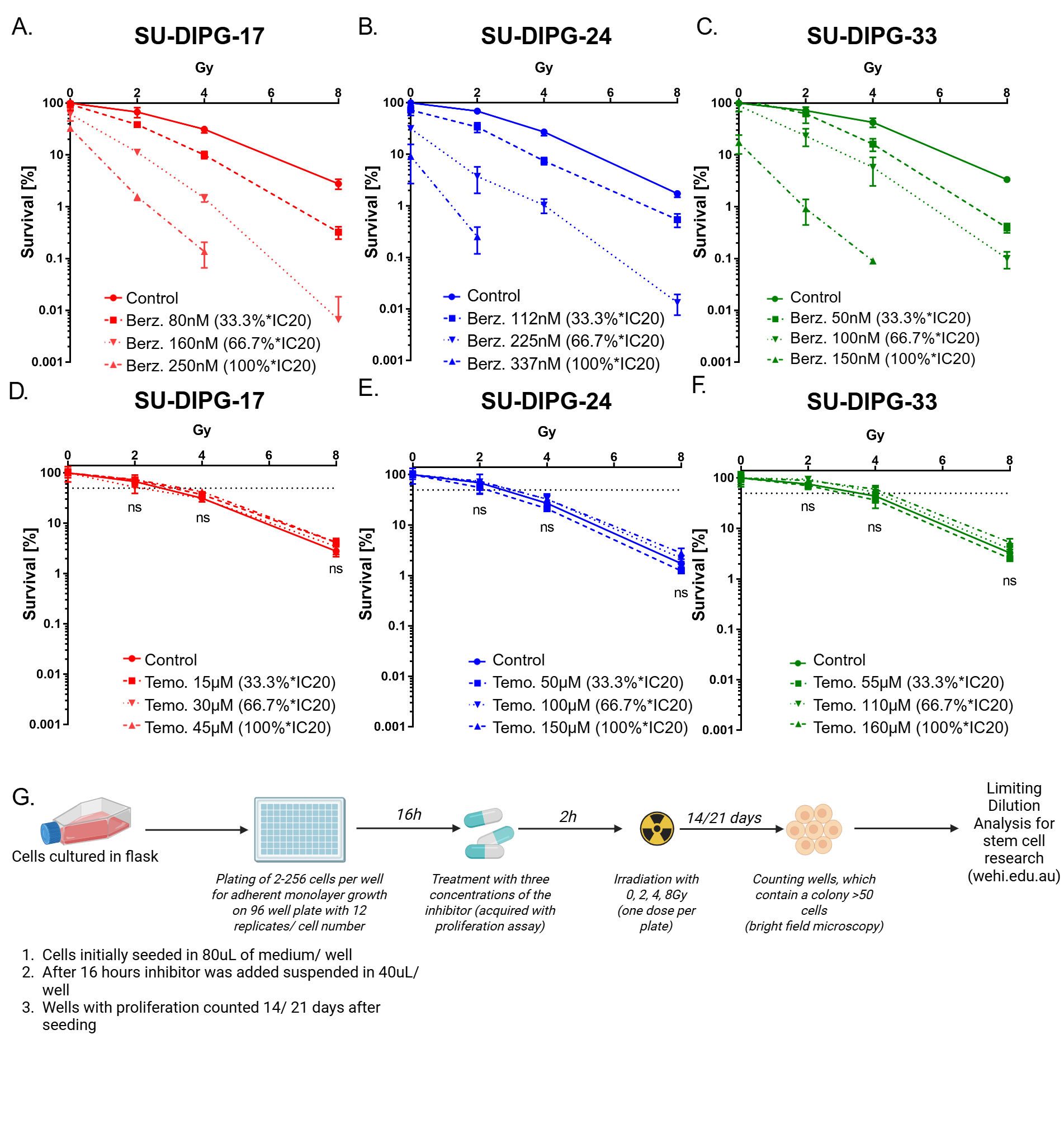
***Supplementary Figure 3. Berzosertib Enhances the long-term Cytotoxic Effect of Ionizing Irradiation in Diffuse Midline Glioma H3K27-altered:*** *Limiting-Dilution-Assay results for SU-DIPG-17 (A), SU-DIPG-24 (B) and SU-DIPG-33 (C) treated with berzosertib (Berz.) 2 hours prior to irradiation normalized to 0 Gy without berzosertib. The applied concentrations have been: 80 nM (33.3%* IC20), 160 nM (66.7%* IC20) and 250 nM (100%*IC20) for SU-DIPG-17, 112.5 nM (33.3%* IC20), 225 nM (66.7%*IC20) and 337.5 nM (100%*IC20) for for SU-DIPG-24 and 50 nM (33.3%* IC20), 100 nM (66.7%*IC20) and 150 nM (100%*IC20) for SU-DIPG-33 respectively. Error bars show standard deviation. Limiting-Dilution-Assay results for SU-DIPG-17 (D), SU-DIPG-24 (E) and SU-DIPG-33 (F) treated with temozolomide (Temo.) 2 hours prior to irradiation. The applied concentrations have been: 15 µM (33.3%* IC20), 30 µM (66.7%* IC20) and 45 µM (100%*IC20) for SU-DIPG-17, 50 µM (33.3%* IC20), 100 µM (66.7%*IC20) and 150 µM (100%*IC20) for for SU-DIPG-24 and 55 µM (33.3%* IC20), 110 µM (66.7%*IC20) and 160 µM (100%*IC20) for SU-DIPG-33 respectively. Survival was normalized to 0 Gy for each temozolomide concentration separately. Dotted line marks 50% survival. Statistical analysis was performed using paired t-Test for log-transformed probability values. Error bars show standard deviation. (G) Visualized protocol of the Limiting-Dilution-Assay; Cells cultured in flasks were plated in concentrations of 2-256 cells/well in 96-well plates. Each plate was used for one treatment condition. 16 hours after plating each plate was treated with one concentration of berzosertib or temozolomide or with DMSO control. Two hours after drug treatment the cells were irradiated with 0, 2, 4 or 8 Gy. After seeding for 14 days for SU-DIPG-17 or SU-DIPG-33 and for 21 days for SU-DIPG-24 the wells containing a colony were counted with brightfield microscopy. Using the ELDA-software, stem-cell-frequency survival was calculated for each condition (*[*http://bioinf.wehi.edu.au/software/elda/*](http://bioinf.wehi.edu.au/software/elda/)*). The image in G) was created with BioRender.com.*

**Supplementary Figure 4. Berzosertib suppressed spheroid growth in combination with irradiation** **(Related to Figure 4)**


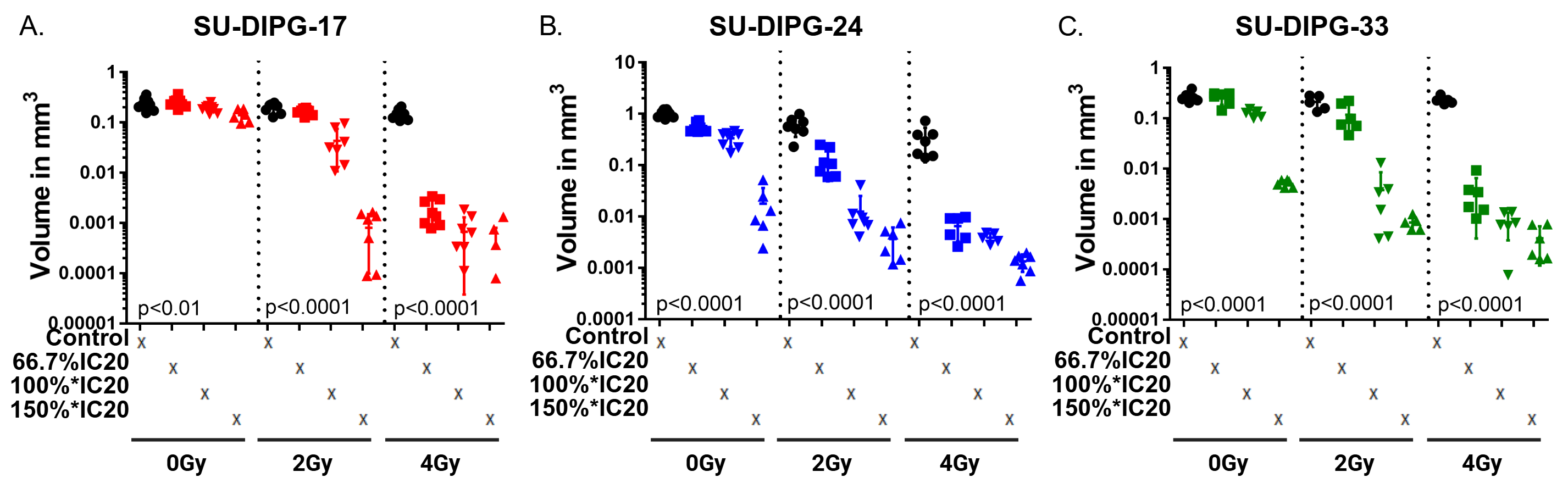


***Supplementary Figure 4. In combination with irradiation, berzosertib suppresses spheroid growth:*** *Spheroid volume at the end of measurement for the three cell lines: SU-DIPG-17 (A), SU-DIPG-24 (B) and SU-DIPG-33 (C), each dot represents a single spheroid. The applied concentrations have been: 160 nM (66.7%* IC20), 250 nM (100%*IC20) and 360 nM (150%*IC20) for SU-DIPG-17, 225 nM (66.7%* IC20), 337.5 nM (100%*IC20) and 500 nM (150%*IC20) for SU-DIPG-24 and 100 nM (66.7%* IC20), 150 nM (100%*IC20) and 225 nM (150%*IC20) for SU-DIPG-33 respectively. Significance was tested with One-Way ANOVA following post-hoc Tukey-test, for each cell-line and irradiation dose separately. Dotted lines separate different irradiation doses. p-value is displayed in left bottom corner for each irradiation dose. Error-bars show standard deviation.*
